# Exosomal circRNA-100338 promotes hepatocellular carcinoma metastasis via enhancing invasiveness and angiogenesis

**DOI:** 10.1186/s13046-020-1529-9

**Published:** 2020-01-23

**Authors:** Xiu-Yan Huang, Zi-Li Huang, Jin Huang, Bin Xu, Xin-Yu Huang, Yong-Hua Xu, Jian Zhou, Zhao-You Tang

**Affiliations:** 1 Department of General Surgery, Shanghai Jiaotong University Affiliated Sixth People’s Hospital, 600 Yi Shan Road, Shanghai, 200233 People’s Republic of China; 2 Department of Radiology, Xuhui District Central Hospital of Zhongshan Hospital, Fudan University, Shanghai, 200031 People’s Republic of China; 3 Department of Pathology, Shanghai Jiaotong University Affiliated Sixth People’s Hospital, Shanghai, 200233 People’s Republic of China; 4 Department of General Surgery, the Tenth People’s Hospital of Tongji University, Shanghai, 200072 People’s Republic of China; 5 Liver Cancer Institute and Zhongshan Hospital, Fudan University, Shanghai, 200032 People’s Republic of China

**Keywords:** CircRNA-100,338, Hepatocellular carcinoma (HCC), Exosome, Tumor metastasis, Angiogenesis

## Abstract

**Background:**

Exosomes play crucial roles in regulating the crosstalk between normal and cancer cells in the tumor microenvironment, and in regulating cancer proliferation, migration and invasion through their cargo molecules.

**Methods:**

We analyzed the pro-invasiveness of exosomal circRNA-100,338 in HCC using the transwell invasion assay. The co-culture of human umbilical vein endothelial cells (HUVEC) and exosomes derived from HCC cell lines were used to evaluate the impact of HCC derived exosomes on HUVEC. Nude mice models were used to validate the findings in vitro. Clinically, quantitative RT-PCR was used to quantify the expression of serum exosomal circRNA-100,338 in HCC patients at both pre-surgery within one week and post-surgery within three weeks.

**Results:**

We aim to investigate the pro-invasive role of exosomal circRNA-100,338 in HCC metastasis. We for the first time demonstrated that circRNA-100,338 was highly expressed in both highly metastatic HCC cells and their secreted exosomes. The transwell invasion assay showed that the overexpression or knockdown of exosomal circRNA-100,338 significantly enhanced or reduced the invasive abilities of HCC cells. Subsequently, in vitro and in vivo assays showed that exosomal circRNA-100,338 affected the cell proliferation, angiogenesis, permeability, and vasculogenic mimicry (VM) formation ability of human umbilical vein endothelial cells (HUVEC), and tumor metastasis. Furthermore, we also observed that the persistent high expression of exosomal circRNA-100,338 in serum of HCC patients who underwent curative hepatectomy may be a risk indicator of pulmonary metastasis and poor survival.

**Conclusions:**

Our findings indicated that metastatic ability of HCC cells could be enhanced by transferring exosomal circRNA-100,338 to recipient HUVECs, which could affect proangiogenic activity by regulating angiogenesis.

## Background

Hepatocellular carcinoma (HCC) is the leading cause of cancer mortalities worldwide [1]. Over the past decades, a number of studies had been conducted to explore the molecular mechanisms underlying the pathogenesis of HCC and they have revealed that gene mutations, epigenetic alterations, and dysregulation of coding or non-coding genes were involved in regulating HCC progression. However, the morbidity and mortality of HCC were still high. Widespread metastases remain to be a major challenge for HCC therapy and contributed to the poor prognosis of HCC [2, 3]. Therefore, identifying novel regulators related to HCC tumorigenesis, progression and metastasis is still an urgent need.

Circular RNAs (circRNAs) are a type of naturally occurring RNAs which are synthesized by “head to tail” splicing of coding or non-coding RNAs (ncRNAs) [4]. circRNAs were identified to be important regulators in human cancers. In HCC, we, together with other research teams [5–7], have identified that a series of circRNAs were dysregulated in cancer samples and associated with tumor progression, which may serve as promising biomarkers for cancer. CircRNAs are involved in regulating multiple cancer-related biological processes and pathways, including cell growth [8], metastasis [9], and apoptosis [10]. For example, circRNA cSMARCA5 can suppress cell metastasis by binding to miR-17-3p to promote TIMP3 expression in HCC [11]. Circ-CDYL interacts with HDGF and HIF1AN to regulate HCC stemness and growth [6]. We previously identified a series of dysregulated circRNAs in HCC and focused on examining the roles of circRNA-100,338 in HCC [5, 12]. We have demonstrated that circRNA-100,338 is overexpressed and associated with mTOR signaling pathway [5] and poor prognosis [12] in HCC. Of note, circRNAs can be detected in blood and urine samples of patients, suggesting that circRNAs may be a type of non-invasive markers for human cancer diagnosis [4]. However, the molecular functions and prognostic value of circRNA-100,338 remains to be further investigated.

Exosomes, a type of extracellular vesicles (30–100 nm), were released from living cells and could be transported to adjacent cells or distant cells [13]. Emerging studies had demonstrated that exosomes played a crucial role in regulating the tumor-normal communication in the tumor microenvironment and thus were involved in regulating multiple cancer-related biological processes, such as cell proliferation, angiogenesis and metastasis [14, 15]. Recently, exosome-mediated transfer of circRNAs is revealed to be a novel mechanism in cancer progression. For instance, Zhang et al. have reported that exosomal circRNAs derived from gastric tumor promotes white adipose browning by targeting the miR-133/PRDM16 pathway [16].

This present study for the first time revealed that exosomal circRNA-100,338 was excessively expressed in highly metastatic HCC cells compared with low metastatic HCC cells. Exosomal circRNA-100,338 enhanced the metastatic ability of HCC cells and stimulated angiogenesis of human umbilical vein endothelial cells (HUVECs). Moreover, we provided clinical evidence that exosomal circRNA-100,338 could be a potential biomarker for HCC. This study provided a novel mechanisms focusing on exosomal circRNA-100,338 to explain the crosstalk between HCC cells and endothelial cells, which promoted angiogenesis and cancer metastasis.

## Material and methods

### HCC cell line and cell culture

The HCC cell lines were cultured following procedures stated in our previous reports [5, 12]. Briefly, the noninvasive human liver cell line of L02 (normal), human HCC cell lines of Hep3B with low invasiveness, and highly invasive HLE, Huh7, BEL7402, SMCC7721, MHCC97L, MHCC97H, HCCLM3 and HCCLM6 were prepared in this study, which were widely used in previous studies [17, 18]. HUVECs were obtained from the American Type Culture Collection (ATCC, Manassas, VA, USA) and grown in RPMI-1640 medium (Gibco-BRL, Gaithersburg, MD, USA) supplemented with 10% fetal bovine serum (HyClone, Logan, UT, USA) in a humidified incubator containing 5% CO_2_ at 37 °C. In all experiments, cells were treated without antibiotics.

### Patients, clinical specimens and follow-up

Informed consent was obtained from each patient, and the Research Ethics Committee of Hospital approved all aspects of this study. The inclusion criteria for 39 patients in this study were (a) patients with hepatitis B from 2016 to 2019; (b) pathologically proven HCC based on WHO criteria; (c) no anticancer treatment prior to hepatectomy and 3 weeks post-operation; (d) exosomes from patient with HCC were used after quality control; (e) availability of frozen biopsy and/or resected lung metastatic HCC tissues; and (f) availability of follow-up data. HCC patients with hepatectomy were followed up every 3 months until June 2019 by monitoring serum AFP levels, abdominal ultrasonography, chest X-ray or computed tomography depending on the patient’s condition. HCC tissues, lung metastatic nodules or pulmonary puncture specimens, plasma exosomes were obtained from the Hospital Clinic for further examination. General data, metastatic characteristics, pathologic characteristics and survival were compared among the groups.

### Cell proliferation assay

The cell proliferation assay was conducted using MTT (3-(4,5-dimethylthiazol-2-yl)-2,5-diphenyltetrazolium bromide) assay according to previous studies [5, 12]. The results were read on a multiwell scanning spectrophotometer. The absorbance values were measured at a wavelength of 450 nm (with a reference of 630 nm).

### Immunohistochemistry (IHC)

IHC was performed as previously described [5, 12]. Primary antibodies (Santa Cruz, diluted 1:100) of CK, TTF-1, Napsin A, Hep Par-1, Villin and Glyp-3 were prepared for lung metastases confirmation, according to the manufacturer’s instructions. A positive reaction of IHC was indicated by a reddish-brown precipitate in the nucleus and cytoplasm. Primary antibodies were replaced by PBS for negative controls. Microvessel density (MVD, using CD34 immunostaining) was counted [19]. Staining for Ki67 tissue expression was performed using the primary anti-Ki67 antibody (1:50, Tokyo, Japan). The Ki67 was calculated for each sample as the percentage of positively stained tumor cells among all counted tumor cells [20]. All slides were independently assessed by two board-certified pathologists who were blinded to the experiment. Any difference in the analysis was resolved by consensus.

### Isolation of exosomes from medium and plasma

The present study isolated exosomes in medium according to previous reports [21]. Briefly, the collected medium was centrifuged at 300 g for 10 min at 4 degree to remove the cell pellet. Then, the supernatant was centrifuged at 2000 g for 10 min at 4 degree to remove the dead cells. Then, the supernatant was centrifuged at 10000 g for 10 min at 4 degree to remove the cell debris. Finally, the supernatant was centrifuged at 110000 g for 2 h at 4 degree to obtain a precipitate which was an isolated exosomes. Exosomes were then re-suspended in pre-cooled PBS. The present study used a ZetaView particle tracker (ParticleMetrix, Germany) to detect the concentration and size of exosomes.

### Transmission electron microscopy assay

Transmission electron microscopy assay was conducted according to a previous report [22]. Briefly, the exosome pellets were suspended in PBS, fixed with 4% paraformaldehyde and applied to a Formvar/carbon film-coated transmission electron microscope grid (Alliance Biosystems, Inc., Osaka, Japan). Subsequently, samples were fixed by incubation with 1% glutaraldehyde, contrasted with 1% uranyl acetate, embedded and polymerized in epoxy resin, subsequently observed under a Hitachi H-7650 transmission electron microscope (Hitachi, Ltd., Tokyo, Japan).

### Transfection

We knocked down [5] and overexpressed [12] circRNA-100,338 in HCC cell lines according to our previous reports.

### RNA isolation and quantitative RT-PCR

RNA isolation and quantitative RT-PCR were conducted according to our previous reports [5, 12]. Primers of hsa_circRNA-100,338 and GAPDH were as follows: GAPDH_F: 5′-GGGAAACTGTGGCGTGAT-3′, GAPDH_R: 5′-GAGTGGGTGTCGCTGTTGA-3′, circRNA-100,338_F:5′-AAAAGCAAGCAGTGCCCATA-3′, circRNA-100,338_R:5′-GCTCGAATCAGGTCCACCA-3′.

### Western blotting

Western blotting was conducted to detect the protein levels of CD63 (1:1000, SBI), CD81 (1:1000, Proteintech), CD9 (1:500, Proteintech) and GAPDH (1:1000, Proteintech) according to our previous reports [5, 12].

### Mice grouping and treatment

Male athymic BALB/c nu/nu mice of 18–20 g at 5 weeks’ age were obtained from the Shanghai Institute of Materia Medica, Chinese Academy of Science. All mice were handled according to the recommendations of the National Institutes of Health Guidelines for Care and Use of Laboratory Animals. The experimental protocol was approved by the Shanghai Medical Experimental Animal Care Committee. Human HCC tumor models produced by MHCC97H were established in nude mice by orthotopic inoculation, as described in our previous publications [23–25]. Briefly, the left lobe of the liver was exposed under anesthesia, and part of the liver surface was mechanically injured with scissors. A piece of MHCC97H tumor tissue (size 2 × 2 × 2 mm) was fixed within the liver tissue. Therapy started on day 1 after HCC tissues implantation. Sixty nude mice randomized into 4 groups were used in this study:

siNC-exo group (*n* = 15): Each mouse received intravenous injection of 100 μL exosomes (1 μg/μL, exosomes derived from MHCC97H cells of control group) into caudal vein once a week and was injected subcutaneously with sterile saline water (NS, 100 μL) daily.

siCIRC-exo group (*n* = 15): Each mouse received intravenous injection of 100 μL exosomes (1 μg/μL, exosomes derived from MHCC97H cells of siCIRC group) into caudal vein once a week and was injected subcutaneously with sterile saline water (NS, 100 μL) daily.

siNC-exo + IFN-alpha group (*n* = 15): Each mouse received intravenous injection of 100 μL exosomes (1 μg/μL, exosomes derived from MHCC97H cells of control group) into caudal vein once a week and was injected subcutaneously with 100 μL of IFN-alpha (IFNα, 7.5 × 10^6^ U/kg/d/mouse) daily [26].

siCIRC-exo + IFN-alpha group (*n* = 15): Each mouse received intravenous injection of 100 μL exosomes (1 μg/μL, exosomes derived from MHCC97H cells of siCIRC group) into caudal vein once a week and was injected subcutaneously with 100 μL of IFN-alpha (IFNα, 7.5 × 10^6^ U/kg/d/mouse) daily.

Five weeks later, 5 mice randomly selected from each group were humanely killed by cervical dislocation 48 h after the final treatment. The remaining 10 mice of each group were maintained on the designated therapies until death to determine their lifespan. Samples were collected to detect exosomal circRNA-100,338, lung metastases, MVD, Ki67 and MMP9 protein levels. Tumor volume was estimated by the formula *V* = *π*/6 × *a*^2^ × *b*, where *a* was the short and *b* was the long tumor axis.

### Hematoxylin and eosin (H&E)

Hematoxylin and eosin stains were conducted according to our previous reports [27].

### The enzyme-linked immunosorbent assay (ELISA) for MMP9

The levels of the MMP9 were measured using ELISA kits from R&D (MN, USA) according to the manufacturer’s instructions. The assays were conducted in triplicate.

### Gelatin zymography for MMP9 and MMP2

Gelatin zymography for MMP9 and MMP2 were performed as previously described [28, 29] with modifications. Briefly, 30 μg of protein were loaded in 8% polyacrylamide gels co-polymerized with 0.1% gelatin (Merck™) acting as the substrate for the enzymes. After electrophoresis, the gels were washed twice in 2.5% Triton X-100 to remove sodium dodecyl sulfate and further washed in 50 mM Tris-HCl pH 8.0. Gels were incubated for the following 20 h in an activation buffer (50 mM Tris-HCl supplemented with 5 mM CaCl2). Gels were stained with Coomassie brilliant blue R-250 and de-stained with 20% methanol and 10% acetic acid in distilled water until the clear bands had been visualized. MMP activity was determined by densitometry using Quantity One 1-D Analysis Software (Bio-Rad Laboratories, CA, USA).

### Transendothelial invasion assay

Transendothelial invasion assay was performed to detect the GFP-expressing hepatoma cells that invaded through HUVEC monolayers without or with exosome treatment according to a previous report [30].

### Tube formation assay

Tube formation assay was performed to assess the effect of exosomal circRNA-100,338 on angiogenesis. Growth factor-reduced Matrigel (BD Biosciences, San Jose, CA, USA) was placed in 48-well plates. HUVECs were first incubated with serum-free medium for 12 h and then transferred onto the 48-well plates precoated with Matrigel. After incubation for 10 h, tube formation was examined in photographs taken under a microscope. The total tube length was determined by measuring the branches of blood vessels using ImageJ software.

### Exosome labelling and tracking

Exosome labelling and tracking was conducted according to a previous report [31]. Red dye PKH26 kit (Sigma-Aldrich, USA) was used to track exosomes according to the manufacturer’s protocol. The labelled exosomes were added to HUVECs and incubated for 6 h.

### Pulldown assay and mass spectrometry

RNA pulldown and mass spectrometry were performed as described before [32]. Precipitated components were separated using SDS-PAGE, followed by silver staining [33]. Differential bands were cut for mass spectrometry. Each assay was performed in triplicate.

### In vitro endothelial permeability assay

The in vitro endothelial permeability was assessed by quantifying the amount of rhodamine B isothiocyanate dextran (rhodamine-dextran, average MW = 70,000; Sigma-Aldrich) that passed through the endothelial monolayers without or with exosome treatment. The primes for circRNA_100,338-P and circRNA_N-P were CTCAACATTCACGTGGTTCCACAAACTTCTCACCATTCTGCT and AAAAAAAAAAAAAAAAAAAAAAAAA, respectively.

### Statistical analysis

All experiments were performed in triplicate, and the results are presented as the mean value ± standard deviation. The data were statistically analyzed using ANOVA. Student’s *t*-test in SPSS statistical software, with *P* < 0.05 considered statistically significant. * indicates *P* < 0.05; ** indicates *P* < 0.01 and *** indicates *P* < 0.001.

## Results

### Characterization of exosomes derived from HCC cell lines

**Fig. 1:**
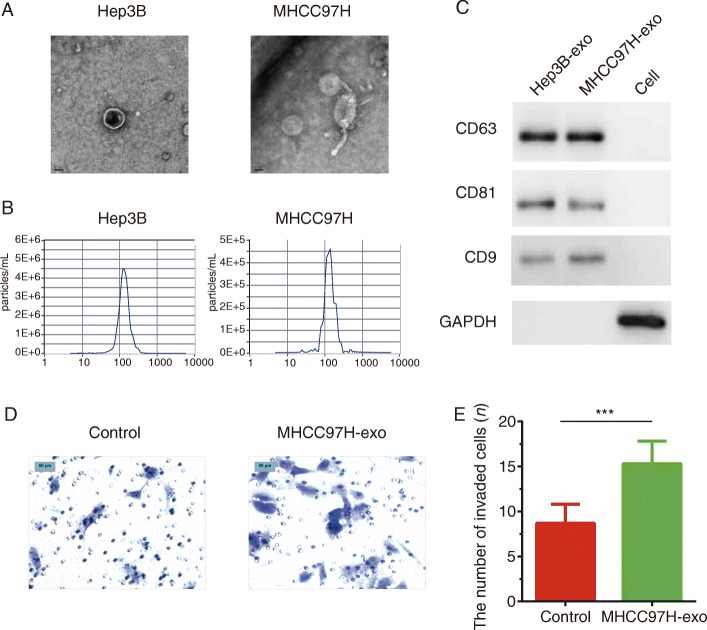
Characterization of exosomes derived from HCC cell lines. **a** TEM image of exosomes isolated from Hep3B and MHCC97H cell lines. **b** The size range of the exosomes isolated from Hep3B and MHCC97H cell lines checked by NAT analysis. **c** WB analysis of exosomal markers, including CD63, CD81 and CD9. **d** Hep3B cells co-cultured with exosomes derived from MHCC97H cells had higher invasive ability than that without MHCC97H exosomes treatment. **e** Histogram plot showed the number of invaded Hep3B cells with or without MHCC97H exosomes (represented by MHCC97H-exo) treatment. Significance was defined as *P* < .05 (*P < .05; ** *P* < .01; *** *P* < .001)

With the validated circular structure and resistance digestion of circRNA-100,338 (See Additional file 1), we focused on exploring the exosome-based mechanisms underlying the metastasis and progression of HCC. To demonstrate the universal expression of circRNA-100,338 in HCC cell lines, we selected normal liver cell line L02, AFP-positive, AFP-negative, highly metastatic potential and lowly metastatic potential HCC cell lines, including HLE, Huh7, Hep3B, BEL7402, SMMC7721, MHCC97L, MHCC97H, HCCLM3, and HCCLM6. Particularly, HLE and Huh7 were AFP-negative cell lines, while the remaining were AFP-positive. The exosomes were isolated and characterized from two representative HCC cell lines, Hep3B and MHCC97H cells, from the nine HBV-positive HCC cell lines with varied metastatic potentials. Notably, MHCC97H and Hep3B were characterized as relatively high and low metastatic potential, in which, the circRNA-100,338 was highly and lowly expressed, respectively [12]. Transmission electron microscopy analysis revealed that exosomes derived from both cell lines showed a round-shaped appearance (Fig. 1a). The nanoparticle tracking analysis (NTA) showed that the size of these exosomes came from a similar distribution with the peak size range about 80–135 nm (Fig. 1b). Western blot analysis confirmed the presence of CD63, CD81, and CD9, which were reported as exosomal markers [34] (Fig. 1c). These results showed the exosomes were successfully isolated from Hep3B and MHCC97H cell lines.

Given that tumor-derived exosomes had been reported to regulate cancer metastasis [35], we hypothesized that exosomes derived from high-metastatic HCC cells might enhance invasiveness of lowly metastatic HCCs. Transwell invasion assay showed that Hep3B cells co-cultured with exosomes derived from MHCC97H cells had higher invasion than the Hep3B cells without MHCC97H exosomes co-incubation (Fig. 1d-e, Additional file 2). Accordingly, more invaded HCC cells were observed in Hep3B cells co-cultured with MHCC97H exosomes (Fig. 1e, *P* < 0.001, Additional file 2). The enhanced invasive ability by MHCC97H exosomes in Hep3B gave us a hint that exosomes played a regulatory role in HCC metastasis.

### High expression of exosomal circRNA-100,338 affects invasive ability of HCC

**Fig. 2:**
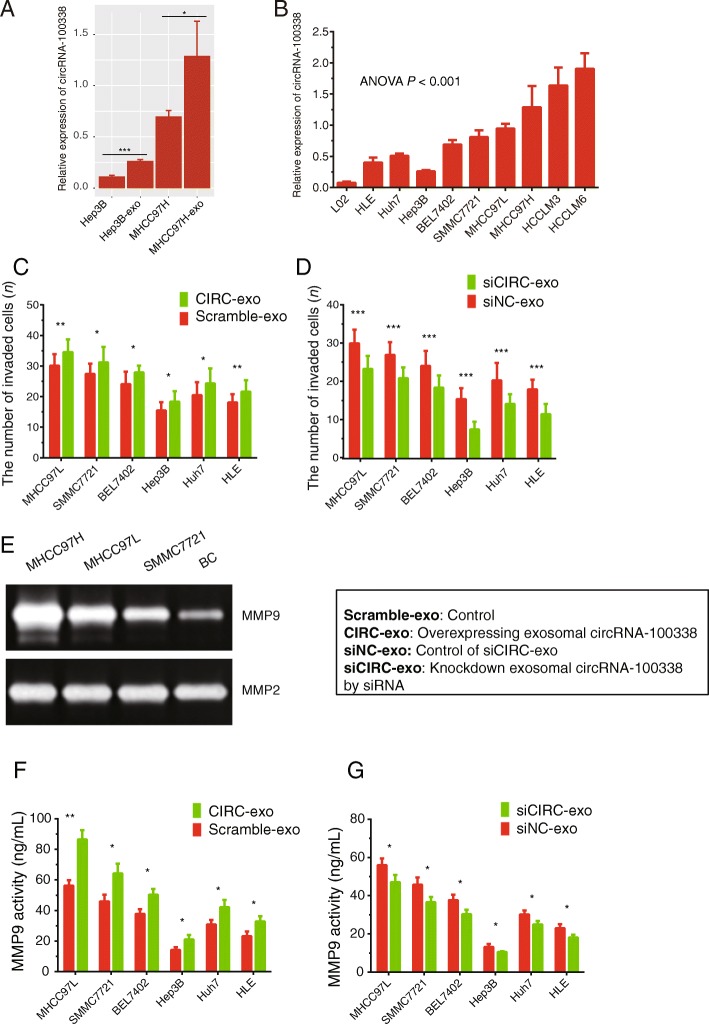
High expression of exosomal circRNA-100,338 affects HCC cell invasion. **a** qRT-PCR analysis of circRNA-100,338 expression in HCC cells (Hep3B and MHCC97H) and in their secreted exosomes (Hep3B-exo and MHCC97H-exo). **b** qRT-PCR analysis of exosomal circRNA-100,338 expression in a series of HCC cell lines with distinct metastatic potential, including HLE, Huh7, Hep3B, BEL7402, SMMC7721, MHCC97L, MHCC97H, HCCLM3, and HCCLM6, and a normal liver cell line, L02. **c** The exosomes derived from circRNA-100,338 overexpressing (CIRC-exo) MHCC97H cells promoted the invasive ability of MHCC97L, SMMC7721, BEL7402, Hep3B, Huh7, and HLE cells. **d** The exosomes derived from circRNA-100,338 knockdown (siCIRC) MHCC97H cells suppressed the invasive ability of MHCC97L, SMMC7721, BEL7402, Hep3B, Huh7, and HLE cells. The control exosomes for CIRC-exo and siCIRC were labeled as Scramble-exo and siNC-exo. **e** Gelatin zymography assay showed the activity of MMP9 and MMP2 in Hep3B after treated with the exosomes derived from MHCC97H. **f-g** ELISA assay showed that exosomes derived from circRNA-100,338 overexpressing (CIRC-exo) or knockdown (siCIRC) MHCC97H cells significantly increased (**f**) or decreased (**g**) the expression levels of MMP9 in MHCC97L, SMMC7721, BEL7402, Hep3B, Huh7, and HLE cells. Significance was defined as *P* < .05 (*P < .05; **P < .01; ***P < .001)

As shown in Fig. 2a, both intracellular and exosomal circRNA-100,338 levels were higher in the metastatic MHCC97H than those in Hep3B (Additional file 3). Meanwhile, the present study also showed that exosomal circRNA-100,338 was positively associated with the metastatic ability of HCC (Fig. 2b, Additional file 3). The exosomal circRNA-100,338 was observed to be significantly more abundant in highly metastatic HCCLM6, HCCLM3, and MHCC97H cells than that in lowly metastatic Huh7 and HLE cells and normal liver cell line, L02 (Fig. 2b). These results showed that circRNA-100,338 could be transferred by exosomes, and suggested that exosomal circRNA-100,338 played a potential regulatory role in HCC metastasis.

In order to determine the pro-invasive role of exosomal circRNA-100,338, we assessed the effect of exosomal circRNA-100,338 on the HCC invasiveness using transwell invasion assay. With the successful knockdown or overexpression of exosomal circRNA-100,338 in MHCC97H, the exosomes derived from circRNA-100,338 overexpressing MHCC97H cells promoted the invasive ability of MHCC97L, SMMC7721, BEL7402, Hep3B, Huh7, and HLE by 14.6, 13.9, 15.9, 18.1, 18.5, and 19.6% (Fig. 2c, Additional file 3), respectively. In contrast, exosomes derived from circRNA-100,338 knockdown MHCC97H cells significantly decreased the invasive ability of MHCC97L, SMMC7721, BEL7402, Hep3B, Huh7, and HLE by 22.3, 22.8, 23.9, 51.5, 30.4, and 36.4%, respectively (Fig. 2d, Additional file 3). In addition, since matrix metalloproteinases (MMP), such as MMP2 and MMP9, played crucial roles in promoting metastasis of HCC [36], Gelatin zymography assay for these two proteins showed the activities of MMP9, not MMP2, in Hep3B were increased after samples were treated with the exosomes derived from MHCC97H, which had increased invasive potential (Fig. 2e). ELISA assay showed that exosomes derived from circRNA-100,338 overexpressing or knockdown MHCC97H cells significantly increased or decreased the expression levels of MMP9 in MHCC97L, SMMC7721, BEL7402, Hep3B, Huh7 and HLE cells (Fig. 2f-g, Additional file 3).

### Exosomal circRNA-100,338 regulates HUVEC cell proliferation, angiogenesis, and permeability

**Fig. 3:**
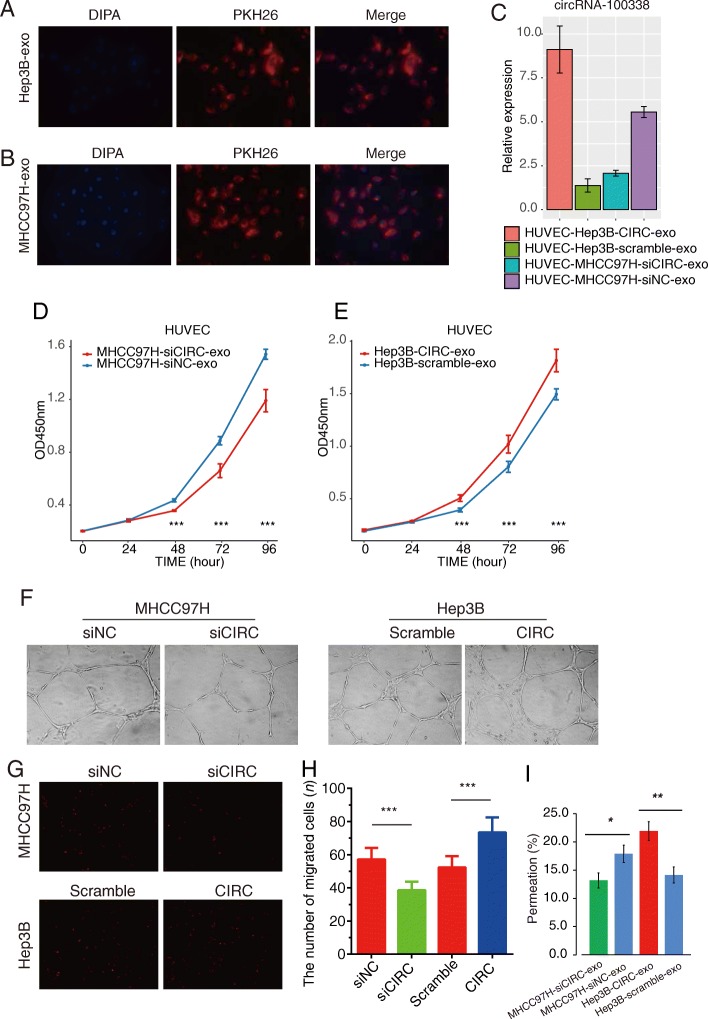
Exosomal circRNA-100,338 regulates HUVEC cell proliferation, angiogenesis, and permeability. **a-b** HUVECs cells after 3 h incubation of exosomes isolated from Hep3B and MHCC97H cells with fluorescently labeled PKH26. Red represents exosomes staining by PKH26, and blue represents nuclear DNA staining by DAPI. **c** qRT-PCR analysis of circRNA-100,338 expression in HUVEC cells after treated with exosomes derived from circRNA-100,338 overexpressing Hep3B (Hep3B-CIRC-exo) or knockdown MHCC97H (MHCC97H-siCIRC-exo) cells, controls of which were labeled as Hep3B-scramble-exo and MHCC97H-siNC-exo. **d-e** CCK-8 assay detected the proliferation rate of HUVEC after treated with exosomes derived from circRNA-100,338 knockdown MHCC97H cells (**d**) and overexpressing Hep3B cells (CIRC-exo) (E). **f** Tube formation of HUVECs after treated with exosomes derived from circRNA-100,338 knockdown MHCC97H cells (siCIRC) and overexpressing Hep3B cells (CIRC). **g** Transwell assay was used to detect the effects of exosomal circRNA-100,338 on the ability to migrate HUVEC cells. **h** Histogram plot showed the number of migrated cells. **i** Moreover, exosomes derived from circRNA-100,338 knockdown MHCC97H cells (siCIRC-exo) and overexpressing Hep3B cells (CIRC-exo) affected the permeability of HUVEC monolayers. Significance was defined as *P* < .05 (*P < .05; **P < .01; ***P < .001)

To investigate the function of exosomal circRNA-100,338, we exposed HUVEC cells to exosomes isolated from Hep3B and MHCC97H cells. As shown in Fig. 3a, fluorescence microscopy assay revealed that HUVECs cells exhibited the uptake of exosomes derived from Hep3B and MHCC97H in the cytoplasm, which were labeled with a red fluorescent dye, PKH26 (Fig. 3a-b). The expression of circRNA-100,338 in HUVEC cells co-cultured with exosomes derived from circRNA-100,338 knockdown MHCC97H cells was significantly lower than that in HUVEC cells co-cultured with controls. In contrast, circRNA-100,338 was significantly upregulated in HUVEC cells co-cultured with exosomes derived from circRNA-100,338 overexpressing Hep3B cells than the controls (Fig. 3c, Additional file 4).

With the significant upregulation or downregulation of circRNA-100,338 in HCC cells, exosomes derived from circRNA-100,338 knockdown MHCC97H cells significantly suppressed the HUVEC cell proliferation after 48 h co-culture (Fig. 3d, Additional file 4). However, exosomes derived from circRNA-100,338 overexpressing Hep3B cells significantly promoted the proliferation of HUVEC cells after 48 h (Fig. 3e, Additional file 4), as compared with their corresponding controls, respectively.

Furthermore, we observed that incubation of conditioned mediums collected from circRNA-100,338 knockdown MHCC97H cells or circRNA-100,338 overexpressing Hep3B cells with HUVEC cells could suppress or enhance the tube formation of HUVEC as compared with their corresponding negative control samples (Fig. 3f). These results suggested that exosomal circRNA-100,338 could promote the HUVEC angiogenesis.

Next, transwell assay was performed to assess the impact of exosomal circRNA-100,338 on HCC cell migration. HUVEC cells were first exposed to exosomes derived from circRNA-100,338 knockdown MHCC97H cells or circRNA-100,338 overexpressing Hep3B cells. The migration of MHCC97H was then tested on the monolayer of HUVEC cells pre-treated by exosomes derived from HCCs. The results showed that exosomes derived from circRNA-100,338 knockdown or overexpressing HCC cell lines significantly suppressed or enhanced the migratory ability of HCC cells (Fig. 3g-h, Additional file 4, Additional file 5). Moreover, the permeation rate of the HCC cells across the HUVEC cells was significantly decreased in circRNA-100,338 knockdown MHCC97H cells, while it was significantly increased in circRNA-100,338 overexpressing Hep3B cells, when compared with control groups (*P* < 0.05, Fig. 3i, Additional file 4). In addition, we also detected the proliferation of HUVEC blank control (HUVEC-BC) and the invaded tumor cells in HUVEC-BC group. Consistently, the proliferation and invasion rates were significantly lower in HUVEC-BC than the HUVECs co-cultured with exosomes (*P* < 0.05), suggesting that the exosomal circRNA-100,338 could regulate HUVEC cell proliferation, angiogenesis, and permeability.

### Exosomal circRNA-100,338 regulates VM formation by regulating VE-cadherin

**Fig. 4:**
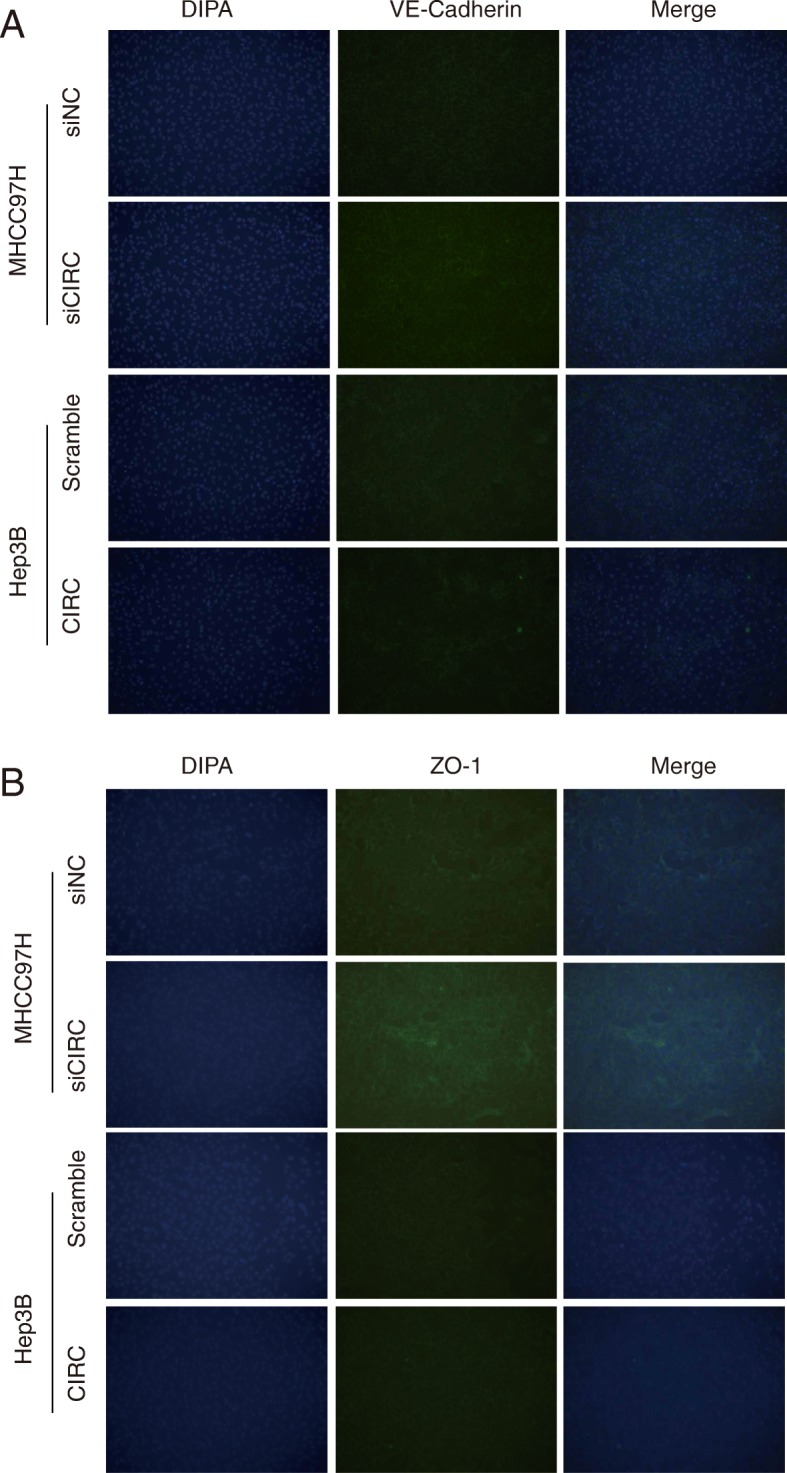
Exosomal circRNA-100,338 regulates VM formation by regulating VE-cadherin. **a** The results showed that exosomes derived from circRNA-100,338 overexpressing Hep3B and knockdown MHCC97H significantly induced and reduced the protein levels of VE-Cadherin in HUEVC cells. **b** The results showed that exosomes derived from circRNA-100,338 overexpressing Hep3B (CIRC) and knockdown MHCC97H (siCIRC) significantly induced and reduced the protein levels of ZO-1 in HUEVC cells

The tight junction protein ZO-1 is often used as an indicator to observe the tight junctional barrier function and permeability of various tissues, and its expression in cancer tissues is lower than that in normal tissues [37]. Vascular endothelial cell cadherin (VE-cadherin) is a key molecule of adhesion junctions between vascular endothelial cells (EC), and its structural and functional abnormalities lead to dissociation of EC adhesion junctions [38]. In order to further evaluate whether exosomal circRNA-100,338 was involved in regulating vasculogenic mimicry (VM) formation in vitro, we first overexpressed and knocked down circRNA-100,338 in Hep3B and MHCC97H cells, respectively, and then collected and incubated their secreted exosomes with HUVECs. Compared with exosomes derived from control Hep3B and circRNA-100,338 knockdown MHCC97H cells, those derived from circRNA-100,338 overexpressing Hep3B and control MHCC97H cells had lower protein expression of VE-Cadherin and ZO-1 in HUEVC cells (Fig. 4a-b, Additional file 5), respectively, suggesting that exosomal circRNA-100,338 could disrupt the tight junctions between HUEVC cells, thus promoting vascular endothelial cell permeability.

### Exosome-delivered circRNA-100,338 significantly promoted HCC progression in vivo

**Fig. 5:**
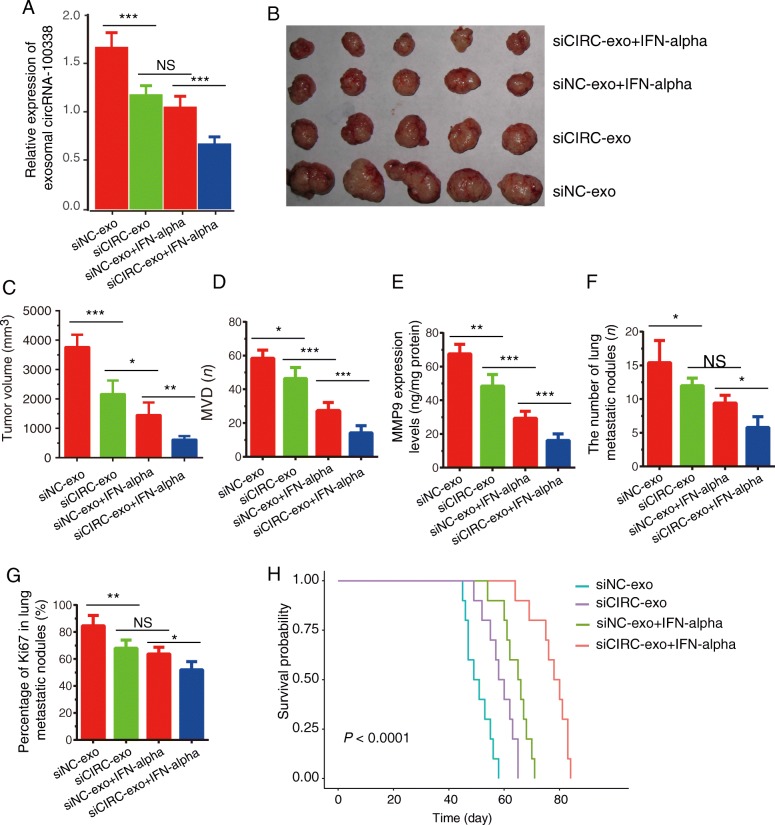
Exosome-delivered circRNA-100,338 significantly promoted HCC progression in vivo. **a** The relative expression of exosomal circRNA-100,338 in the serum of nude mice models. **b** Images of tumors in each group (*n* = 5). (C-G) We calculated the tumor volumes (**c**), microvessel density (**d**), MMP9 expression levels (**e**), the number of lung metastatic nodules (**f**) and positive rate of Ki67 in lung metastatic nodules (**g**) in each group. **h** The survival time of mouse treated control exosome group (siNC-exo), circRNA-100,338 knockdown exosome group (siCIRC-exo), IFNα group (siNC-exo + IFN-alpha) and both circRNA-100,338 knockdown exosome and IFNα group (siCIRC-exo + IFN-alpha). Significance was defined as *P* < .05 (*P < .05; **P < .01; ***P < .001)

We further examined the possibility of exosome-delivered circRNA-100,338 being involved in HCC progression in vivo. We found that the siNC-exo group had the highest expression of exosomal circRNA-100,338 in the serum, followed by groups of siCIRC-exo, siNC-exo + IFN-alpha, and siCIRC-IFN-alpha (*P* < 0.001, Fig. 5a, Additional file 6). Interestingly, we found that knockdown of exosomal circRNA-100,338 could significantly suppress tumor growth (Fig. 5b-c), microvessel density (Fig. 5d), MMP9 expression levels (Fig. 5e), and reduce the number of lung metastatic nodules (Fig. 5f) and the positive rate of Ki67 in lung metastatic nodules (Fig. 5g) of the nude mice models (Additional file 6). Previous study had demonstrated that IFN-alpha inhibited angiogenesis and progression of HCC [39]. Consistently, this study also observed that knockdown of circRNA-100,338 combined with IFN-alpha played a synergistic role in reversing exosome mediated tumor progression. In vivo, circRNA-100,338 knockdown markedly prolonged animal survivals when compared with control group, meanwhile, circRNA-100,338 knockdown combined with IFN-alpha had a stronger effect on prolonging animal survivals than treating the mouse with IFN-alpha alone (Fig. 5h). The results showed that circRNA-100,338 knockdown combined with IFN-alpha had a stronger suppressive effect on HCC growth.

### The potential effect of internalized exosomal circRNA-100,338 on angiogenesis in HUVEC

In order to explore the molecular mechanism of circRNA-100,338, we transfected HUVEC cells with biotin labeled circRNA-100,338 probe and negative control probe respectively and carried out RNA pull down assay. Results of sodium dodecyl sulfate–polyacrylamide gel electrophoresis (SDS-PAGE) protein electrophoresis showed that circRNA-100,338 specifically binds four bands, but the control probe cannot bind these proteins. We excised the differential bands for mass spectrometry and detected 661 proteins (Additional file 7, Additional file 8). Interestingly, circRNA-100,338 can bind 14 RNA binding proteins including FUS, IF2B1, IF2B3, IF2B2, NOVA2, RBM39, RBM14, PAIRB, EWS, NOVA1, RBM26, RBM27, RBM10 and RBM15, five transcription factors including T2FA, HLTF, GTF2I, T2EA and BCLF1, and one mRNA decapping enzyme DCP1A. In addition, circRNA-100,338 may bind to tumor suppressor molecule of p53, histone modifying proteins including HDAC1, HDAC2 and HPF1. Particularly, NOVA2, a RNA binding protein regulating the RNA post-transcriptional modification, was reported to regulate vascular development and lumen formation [40], giving us a hint that the internalized exosomal circRNA-100,338 might regulate the angiogenesis by interacting with NOVA2.

### Serum exosomal circRNA-100,338 can predict lung metastasis of HCC patients following curative hepatectomy

**Fig. 6:**
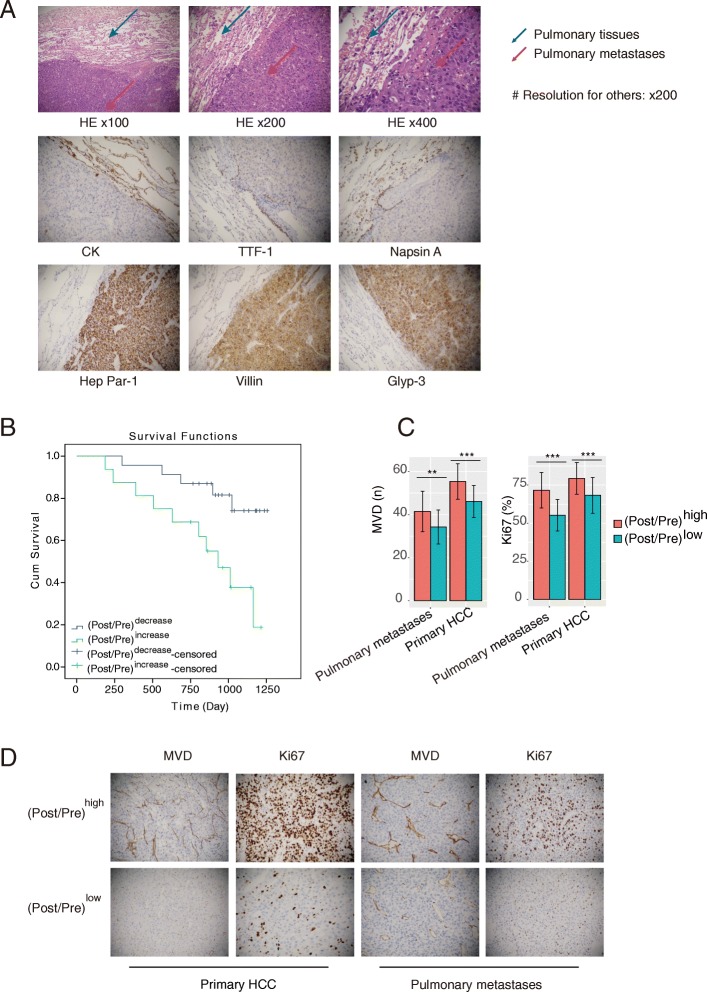
Serum exosomal circRNA-100,338 can predict lung metastasis in HCC. **a** The HE stains showed the presence of nodules in the lung tissues. The lung tissue-specific proteins of CK, TIF-1, and Napsin A and the HCC-specific proteins of Hep Par-1, Villin, and Glyp-3 were only detected in lung and pulmonary metastatic tissues, respectively, indicating that the lung nodules were metastases of HCC. **b** The Kaplan-Meier curves for HCC patients in (Post/Pre)^increase^ and (Post/Pre)^decrease^ groups. **c** Histogram plot and IHC assay (**d**) showed The MVD and positive rates of Ki-67 expression levels were more observed in (Post/Pre)^high^ group than that in (Post/Pre)^low^ group of both primary HCC tissues and pulmonary metastatic tissues. Significance was defined as *P* < .05 (*P < .05; **P < .01; ***P < .001)

To determine whether exosomal circRNA-100,338 can be detected in the circulation, we tested its expression levels in serum of 39 HCC patients, where 13 cases were found to exhibit pulmonary metastasis during follow-up. The lung metastatic nodules were confirmed by pathological examination (Fig. 6a). The expression levels of exosomal circRNA-100,338 were detected in the serum at both one week before the surgery and three weeks after the surgery, during which, the patients did not receive any other anti-tumor treatments. The ratio of its pre-operation expression to post-operation expression was used as a prognostic indicator for HCC. The 39 HCC patients were stratified into two groups ((Post/Pre)^increase^ vs. (Post/Pre)^decrease^, representing the samples with ratio ≥ 1 or <  1, respectively). Consistently, higher rate of pulmonary metastasis was observed in (Post/Pre)^increase^ group (10/16, 62.5%) than that in (Post/Pre)^decrease^ group (3/23, 13.0%, proportion test, *P* = 0.004, Table 1), however, AFP levels of these two groups did not have significant difference (*P* > 0.05) at both pre-surgery and post-surgery points, suggesting that the ratio of Post/Pre was a risk indicator of pulmonary metastasis superior to AFP at the early stage of HCC after curative hepatectomy. Survival analysis of these two groups revealed that patients in (Post/Pre)^decrease^ group exhibited longer overall survival than that in (Post/Pre)^increase^ group (Fig. 6b, *P* = 0.007, 3-year-survival: 18/23 vs. 7/16). Moreover, other prognostic indicators such as TNM stage and vascular invasion were also associated with circRNA-100,338 expression ratio (Table 1).

**Table 1:** Correlation of clinicopathological parameters with the alteration of circRNA_100,338 relative expression level in serum exosomes from HCC patients

Clinicopathological parameters	*n*	CircRNA_100,338 alteration in serum exosomes		*P*
		(Post/Pre)^decrease^ group	(Post/Pre)^increase^ group	
		(< 1.0, *n* = 23)	(≥ 1.0, *n* = 16)	
Age	39			0.688
< 58 y	18	10	8	
≥ 58y	21	13	8	
Gender	39			0.862
Female	14	8	6	
Male	25	15	10	
Viral hepatitis	39			1.000
Type B	37	22	15	
Type B and C	2	1	1	
Cirrhosis	39			0.677
Yes	33	19	14	
No	6	4	2	
AFP level	39			0.939
< 20 ng/mL	10	6	4	
≥ 20 ng/mL	29	17	12	
Satellite	39			0.018
Yes	18	7	11	
No	21	16	5	
TNM stage	39			0.022
I-II	19	15	4	
III-IV_A_	20	8	12	
Vascular invasion	39			0.023
Yes	16	6	10	
No	23	17	6	
γ-Glutamyl transferase	39			0.522
< 51 units/L	17	11	6	
≥ 51 units/L	22	12	10	
Lung metastasis^#^	39			0.004
Yes	13	3	10	
No	26	20	6	

NOTE: HCC, hepatocellular carcinoma; Post/Pre: the ratio of exosomal circRNA-100,338 in HCC serum between one week pre-operation and three weeks post-operation. Significant difference: *P* < 0.05

^#^Lung metastasis was found during follow-up

To further investigate the association of exosomal circRNA-100,338 in serum with MVD and Ki-67 expression of both HCC primary and pulmonary metastatic tissues, we classified the 13 patients with pulmonary metastasis into high ratio ((Post/Pre)^high^, *n* = 8) and low ratio ((Post/Pre)^low^, *n* = 5) groups with the threshold at 1.2 (mean of the ratios). We detected MVD and Ki-67 expression in the primary HCC tissues and lung metastatic tissues from the 13 HCC patients with pulmonary metastasis using IHC. The (Post/Pre)^high^ group had higher MVD and Ki-67 expression than the (Post/Pre)^low^ group in both primary HCC tissues and pulmonary metastatic tissues (*P* < 0.001, Fig. 6c and d, Additional file 9). These results further indicated that high expression of exosomal circRNA-100,338 in serum may be associated with enhanced proliferation and angiogenesis in primary and secondary HCC tissues, and poor prognosis.

## Discussion

The crucial roles of circRNAs in human cancers had been implied by emerging studies [41, 42]. Exosomes can regulate the crosstalk between normal and cancer cells in the tumor microenvironment, cancer proliferation, migration and invasion through their cargo molecules [43–45]. Most recently, exosomal circRNA have attracted increasing interest. For example, exosomal circRNA_100284 promoted liver cancer cell cycle and proliferation through microRNA-217/EZH2 axis [22]. Exosomal circPTGR1 enhanced cancer metastasis in HCC [46]. Exosomal ciRS-133 derived from gastric tumor could sponge miR-133 to promote white adipose browning [16]. CircRNA-100,338 is a novel circRNAs related to the cancer progression. Our previous studies have demonstrated that circRNA-100,338 is overexpressed and associated with mTOR signaling pathway and poor prognosis in HCC [5, 12]. However, the molecular functions of circRNA-100,338 in HCC need to be further investigated. The present study revealed that exosomes derived from high metastatic HCC cells could enhance HCC cell migration, suggesting that exosomes play a regulatory role in HCC metastasis. We then for the first time showed that circRNA-100,338 was highly expressed in both metastatic HCC cells and their secreted exosomes. The transwell invasion assay showed that the overexpression or knockdown of exosomal circRNA-100,338 significantly enhanced or reduced the invasive abilities of HCC cells. Subsequently, our results showed that exosomal circRNA-100,338 affected the cell proliferation, angiogenesis, permeability and VM formation ability of HUVECs. Taken together, these findings indicated that metastatic ability of HCC cells could be enhanced by transferring exosomal circRNA-100,338 to recipient HUVECs via increasing proangiogenic activity.

Emerging studies have demonstrated that angiogenesis played a critical role in the regulation of cancer metastasis [47]. In tumor microenvironment, the endothelial cells and cancer cells can communicate with each other through exosomes, which regulates the angiogenesis and cancel cell progression [48]. We next explored whether HCC derived exosomes and the exosomal circRNA-100,338 were involved in the communication between HUVECs and HCC cells. The results showed that exosomes derived from MHCC97H cells with high metastatic potential had a higher expression of circRNA-100,338 compared with that in Hep3B cells, suggesting that exosomal circRNA-100,338 was involved in regulating HCC metastasis. Furthermore, our results showed that exosomal circRNA-100,338 could significantly promoted HCC cell invasion ability. Moreover, we used exosomes from circRNA-100,338 overexpressing or knockdown HCC cells to treat HUVECs and found that these exosomes could induce or reduce HUVECs cell proliferation, angiogenesis, permeability and VM formation. Finally, we transfected HUVEC cells with biotin labeled circRNA-100,338 probe and negative control probe respectively and carried out RNA pull down assay. Particularly, NOVA2, a RNA binding protein regulating the RNA post-transcriptional modification, was reported to regulate vascular development and lumen formation [40], giving us a hint that the internalized exosomal circRNA-100,338 might regulate the angiogenesis by interacting with NOVA2. The in vivo assays further validated our findings that exosomal circRNA-100,338 promoted HCC metastasis through regulating angiogenesis. These results improved our understanding that exosome-enriched circRNAs were also involved in regulating cancer metastasis.

Alpha-fetoprotein (AFP) is the most widely used marker for HCC diagnosis, and the sensitivity of AFP is as low as about 60% for HCC diagnosis [49]. Specifically, only one of the 13 HCC patients with pulmonary metastasis in this study showed positive AFP within 3 weeks of post-surgery, suggesting that AFP was not sensitive enough to predict the pulmonary metastasis of HCC at the early stage following curative hepatectomy. There is still an urgent need to identify novel biomarkers for HCC. CircRNAs were a type of highly tissue-specific and spatiotemporal-specific molecules, and were reported to be potential biomarkers for multiple human cancers, including HCC [50]. For instance, hsa_circ_0091579 was significantly upregulated in tumor samples and related to poorer prognosis of HCC patients [51]. A recent study showed that the hsa_circ _00520 was associated with relapse-free survival and exhibited relatively high sensitivities and specificities compared with AFP [52]. Notably, circRNAs had been proved to be a type of non-invasive diagnosis markers for human cancers. The present study for the first time showed that exosomal circRNA-100,338 also have the potential prognostic and diagnostic value in HCC. The exosomal circRNA-100,338, the number of MVD, and percentage of positive Ki67 were higher in HCC patients with pulmonary metastasis compared to non-metastatic HCC samples. Moreover, we also found that the change of serum exosomal circRNA-100,338 after the surgery could predict the pulmonary metastasis of HCC, which was more sensitive than AFP in the present study.

In addition, the present study also has some limitations. The lack of detailed molecular mechanism of exosomal circRNA-100,338 is one of the major limitations. Moreover, the clinical significance of the exosomal circRNA-100,338 in serum of HCC patients need to be further investigated in samples of a larger size. It is of great importance for the clinicians to develop anticipative therapeutic strategies, if the diagnostic and prognostic values of exosomal circRNA-100,338 in serum of HCC patients can be validated in HCC cohorts with larger sample size.

## Conclusions

In conclusion, this study for the first time showed that exosomal circRNA-100,338 participated in the regulation of angiogenesis and HCC metastasis. Additionally, we also demonstrated that exosomal circRNA-100,338 was associated with HCC progression in nude mice model. This study provided a novel mechanism regarding the crosstalk between HCC metastasis and angiogenesis mediated by exosomal circRNA-100,338, which greatly improved our understanding of the circRNA-100,338 function.

## Supplementary information

**Additional file 1.** The circular structure and resistance digestion of circRNA-100,338

**Additional file 2: Figure S1E.** The number of invaded cells (*n*)

**Additional file 3: Figure S2A.** Relative expression of circRNA-100,338**, Figure S2B.** Relative expression of circRNA-100,338, **Figure S2C.** The number of invaded cells (*n***), Figure S2D.** The number of invaded cells (*n*), **Figure S2F.** MMP9 activity (ng/mL), **Figure S2G.** MMP9 activity (ng/mL)

**Additional file 4: Figure S3C.** Relative circRNA-100,338 expression, **Figure S3D.** Proliferation of HUVEC cells, **Figure S3E.** Proliferation of HUVEC cells, **Figure S3H.** The number of migrated cells (*n*)

**Additional file 5.** The high resolution version of **Figures 3G and 4**.

**Additional file 6: Figure S5A.** Relative expression of exosomal circRNA-100,338, **Figure S5C.** Tumor volume (cm^3^), **Figure S5D.** MVD (*n*), **Figure S5E.** MMP9 level by ELISA analysis (ng/mg protein), **Figure S5F.** The mean number of lung metastatic nodules (*n*), **Figure S5G.** Percentage of Ki67 in lung metastatic nodules (%)

**Additional file 7.** The result of RNA pull-down experiment.

**Additional file 8.** The proteins interacting with circRNA-100338 in HUVEC.

**Additional file 9: Figure S6C.**

## Data Availability

All remaining data are available within the article or available from the authors upon request.

## Abbreviations

AFP: Alpha-fetoprotein
circRNA: Circular RNAs
ELISA: Enzyme-linked immunosorbent assay
HCC: Hepatocellular carcinoma
HUVEC: Human umbilical vein endothelial cells
IHC: Immunohistochemistry
MMP: Matrix metalloproteinases
MVD: Microvessel density
ncRNA: Non-coding RNAs
NTA: Nanoparticle tracking analysis
RT-PCR: Reverse Transcription-Polymerase Chain Reaction
VM: Vasculogenic mimicry
