# Supplementary material for: Exosomal circRNA-100338 promotes hepatocellular carcinoma metastasis via enhancing invasiveness and angiogenesis
Source: J Exp Clin Cancer Res. 2020 Jan 23;39:20. doi: 10.1186/s13046-020-1529-9 (PMC6979009; doi:10.1186/s13046-020-1529-9)
Supplement: Supplementary file 1 — Additional file 1. The circular structure and resistance digestion of circRNA-100,338 [file 13046_2020_1529_MOESM1_ESM.docx]

**The circular structure and resistance digestion of circRNA-100338**

We performed RNase R (Epicentre Technologies, USA) treatment to degrade linear RNAs. Briefly, 2 μg of total RNA was incubated for 30 min at 37 °C using RNase R (3 U/μg). For Actinomycin D assay, 1×104 MHCC97H cells were seeded into 6-well plate and incubated for 24 h. Then, 2 mg/L Actinomycin D (Sigma, USA) was added into each well for 4, 8, 12 and 24 h respectively. Afterwards, the treated cells were harvested at the indicated time points for qRT-PCR analysis. The expression of circRNA-100338 was not significantly altered by the RNase R treatment, while the linear RNA (termed as 100338mRNA) of the circRNA-100338 host gene *SNX27* was significantly degraded by the RNase R treatment (*P* < 0.001). These results demonstrated that the circRNA-100338 had the ability of resistance digestion by RNase R and stability in HCC cells under treatment with actinomycin D (a transcription inhibitor).

Moreover, in contrast to the linear RNA of *SNX27*, circRNA-100338 was resistant to RNase R, a processive 3’ to 5’ exoribonuclease that degrades linear RNAs but does not digest circRNAs (Figure A). Furthermore, actinomycin D, an inhibitor of transcription, was applied to treat HCC cells, and then RNA was harvested at the indicated time for detection of circRNA-100338 levels. The results showed that circRNA-100338 exhibited a half-life more than 24 hours, whereas the half-life of the linear 100338 mRNA was less than 8 hours (Figure B). Collectively, these data confirmed that circRNA-100338 is an exonic circRNA that is an abundant, circular and highly stable transcript of *SNX27* in HCC cells. In addition, we also designed a specific primer for circRNA-100338, which spanned the backsplice junction, and detected PCR products, suggesting that circRNA-100338 had the circular structure.


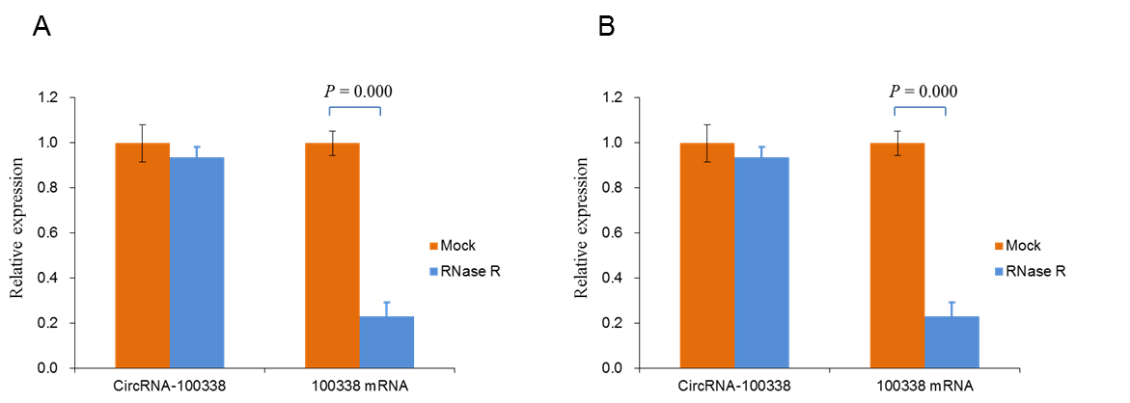


**(A) qRT-PCR analysis was performed to test circRNA-100338 expression in HCC cells with or without RNase R treatment. (B) Total RNA harvested from HCC cells with Actinomycin D treatment at the indicated time points was subjected to qRT-PCR.**
