# Supplementary material for: Exosomal circRNA-100338 promotes hepatocellular carcinoma metastasis via enhancing invasiveness and angiogenesis
Source: J Exp Clin Cancer Res. 2020 Jan 23;39:20. doi: 10.1186/s13046-020-1529-9 (PMC6979009; doi:10.1186/s13046-020-1529-9)
Supplement: Supplementary file 5 — Additional file 5. The high resolution version of Figures 3G and 4. [file 13046_2020_1529_MOESM5_ESM.pptx]

## Slide 1
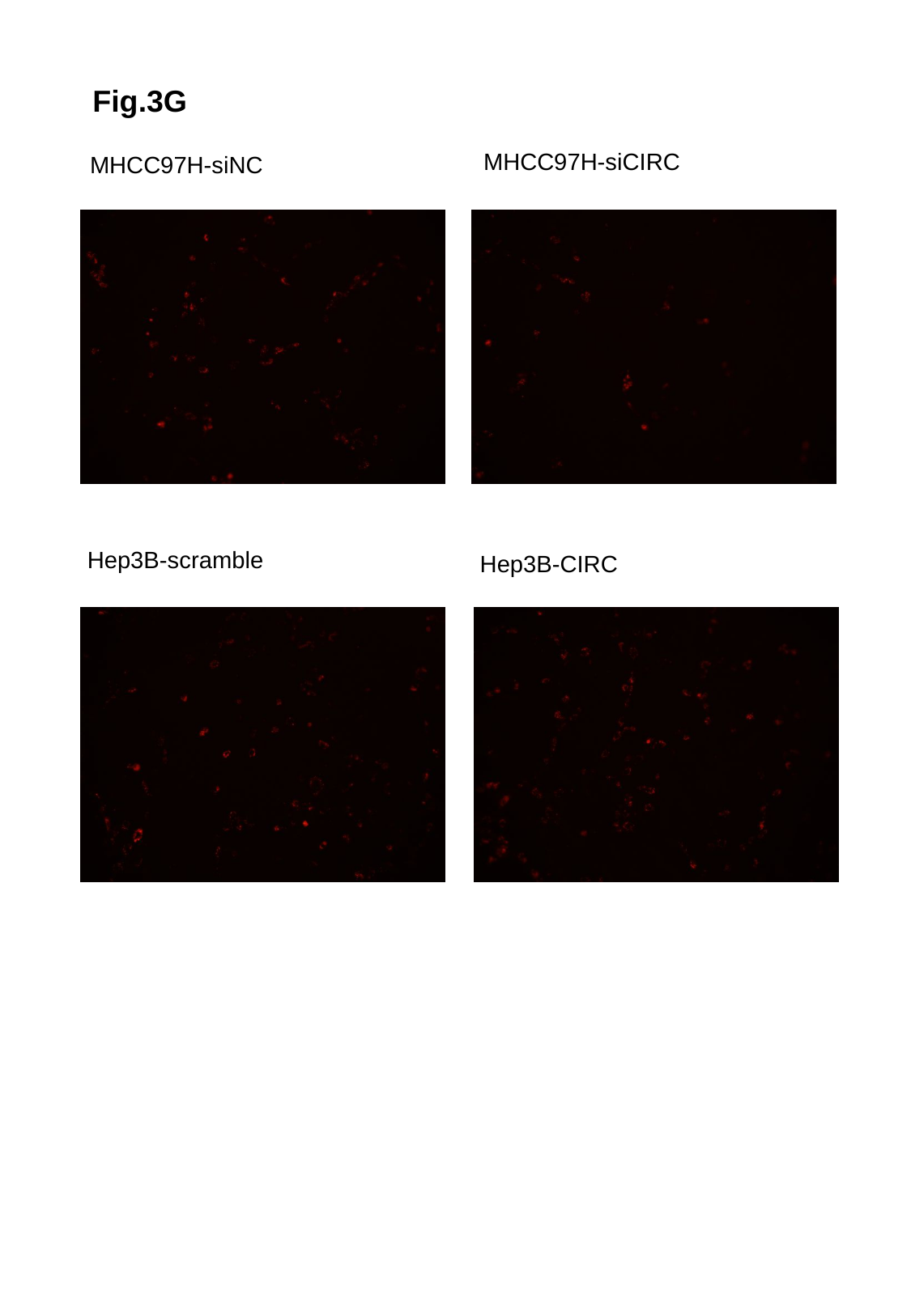

Fig.3G
MHCC97H-siCIRC
MHCC97H-siNC
Hep3B-scramble
Hep3B-CIRC

## Slide 2
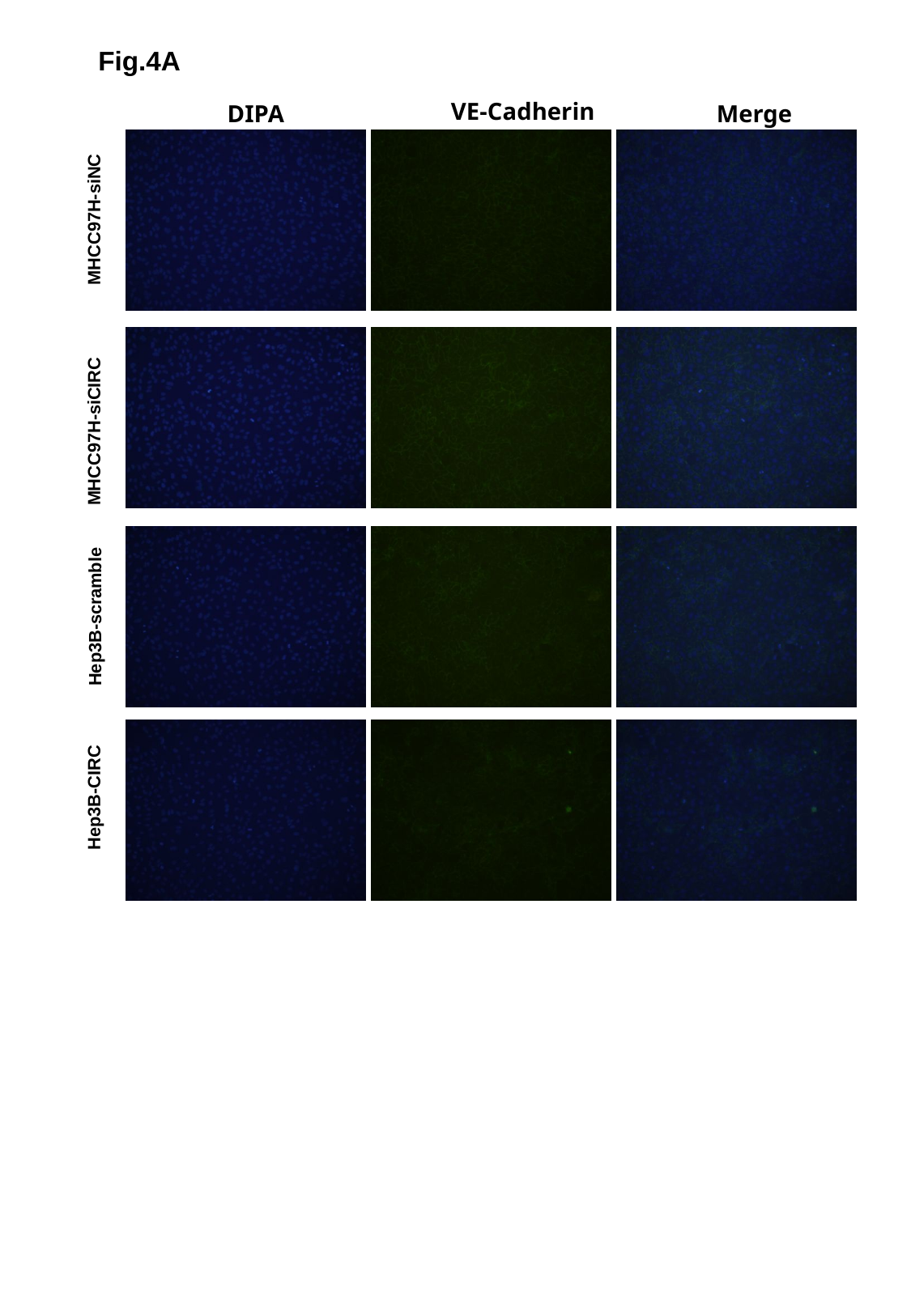

Fig.4A
VE-Cadherin
DIPA
Merge
MHCC97H-siNC
MHCC97H-siCIRC
Hep3B-scramble
Hep3B-CIRC

## Slide 3
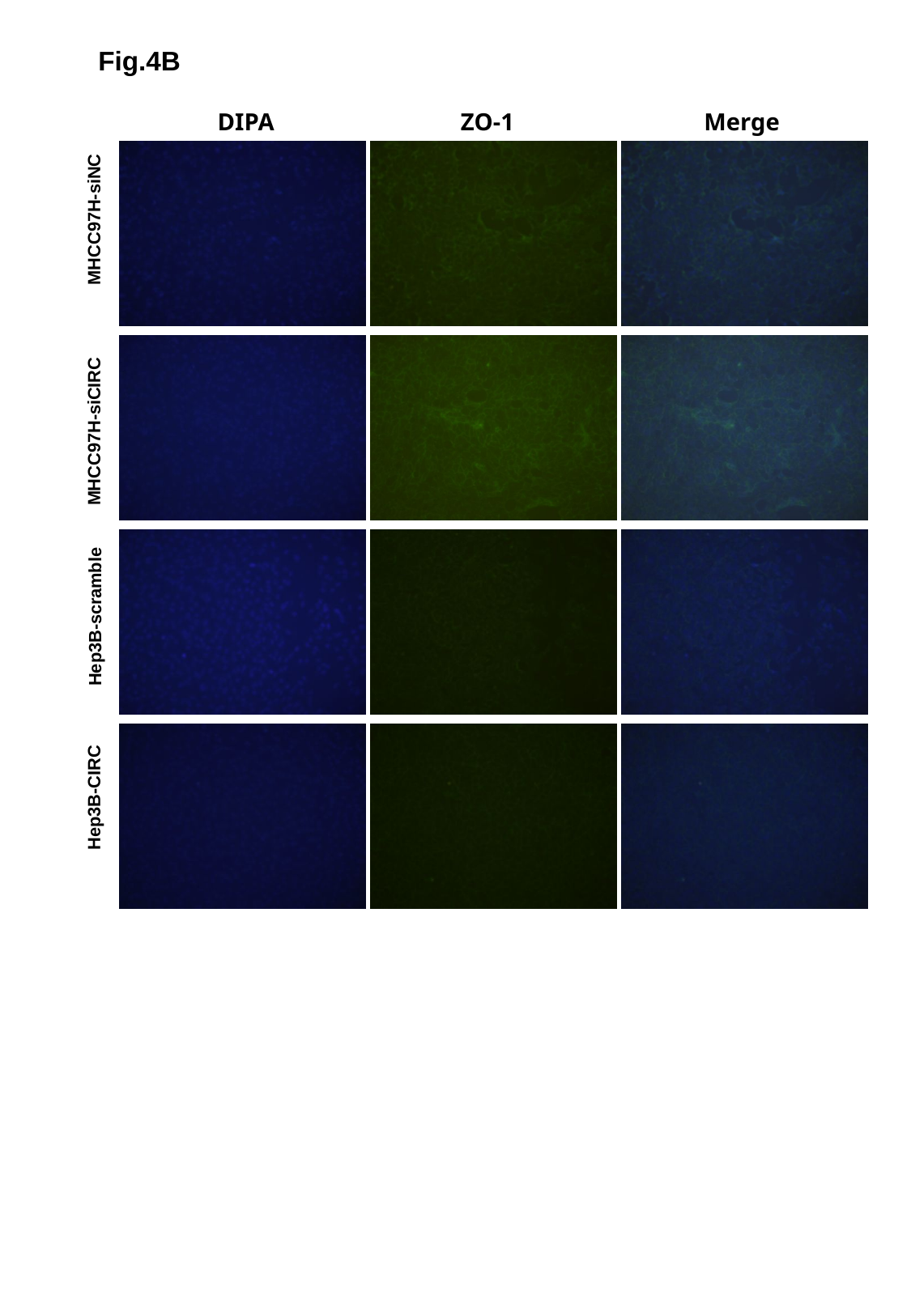

Fig.4B
ZO-1
DIPA
Merge
MHCC97H-siNC
MHCC97H-siCIRC
Hep3B-scramble
Hep3B-CIRC
