# Supplementary figures and images for: Exosomal circRNA-100338 promotes hepatocellular carcinoma metastasis via enhancing invasiveness and angiogenesis
Source: J Exp Clin Cancer Res. 2020 Jan 23;39:20. doi: 10.1186/s13046-020-1529-9 (PMC6979009; doi:10.1186/s13046-020-1529-9)

## Slide 1
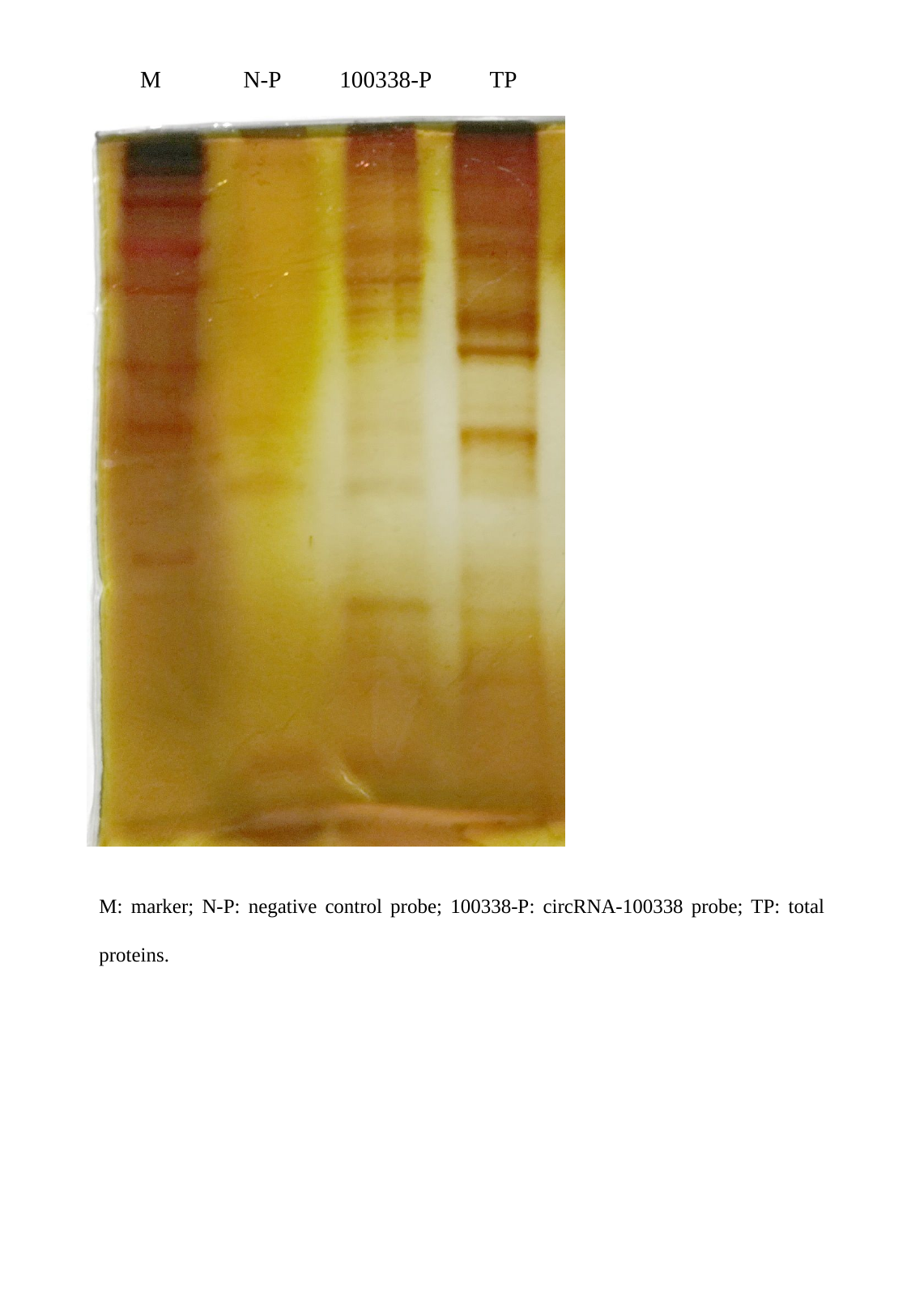

M N-P 100338-P TP
M: marker; N-P: negative control probe; 100338-P: circRNA-100338 probe; TP: total proteins.

Supplement: Supplementary file 7 — Additional file 7. The result of RNA pull-down experiment. [file 13046_2020_1529_MOESM7_ESM.pptx]
